# Lipopolysaccharide Stimulates p62-Dependent Autophagy-Like Aggregate Clearance in Hepatocytes

**DOI:** 10.1155/2014/267350

**Published:** 2014-02-10

**Authors:** Christine Chen, Meihong Deng, Qian Sun, Patricia Loughran, Timothy R. Billiar, Melanie J. Scott

**Affiliations:** ^1^Department of Surgery, University of Pittsburgh, NW607 MUH, 3459 Fifth Avenue, Pittsburgh, PA 15213, USA; ^2^Department of Pathology, University of Pittsburgh, Pittsburgh, PA 15213, USA; ^3^Center for Biologic Imaging, University of Pittsburgh, Pittsburgh, PA 15213, USA

## Abstract

Impairment of autophagy has been associated with liver injury. TLR4-stimulation by LPS upregulates autophagy in hepatocytes, although the signaling pathways involved remain elusive. The objective of this study was to determine the signaling pathway leading to LPS-stimulated autophagy in hepatocytes. Cell lysates from livers of wild type (WT; C57BL/6) mice given LPS (5 mg/kg-IP) and hepatocytes from WT, TLR4ko, and MyD88ko mice treated with LPS (100 ng/mL) up to 24 h were collected. LC3II, p62/SQSTM1, Nrf2, and beclin1 levels were determined by immunoblot, immunofluorescence, and qPCR. Autophagy-like activation was measured by GFP-LC3-puncta formation and LC3II-expression. Beclin1, Nrf2, p62, MyD88, and TIRAP were knocked-down using siRNA. LC3II-expression increased in both liver and hepatocytes after LPS and was dependent on TLR4. Beclin1 expression did not increase after LPS in hepatocytes and beclin1-knockdown did not affect LC3II levels. In hepatocytes given LPS, expression of p62 increased and p62 colocalized with LC3. p62-knockdown prevented LC3II puncta formation. LPS-induced LC3II/p62-puncta also required MyD88/TIRAP signaling and localization of both Nrf2 and NF**κ**B transcription factors to the nucleus to upregulate p62-expression. Therefore, TLR4-activation by LPS in hepatocytes induces a p62-mediated, not beclin1-mediated, autophagy-like clearance pathway that is hepatoprotective by clearing aggregate-prone or misfolded proteins from the cytosol and preserving energy homeostasis under stress.

## 1. Introduction

Autophagy is a protein lysosomal delivery system that breaks down intracellular components and is known to play an essential role in liver physiology and pathology [1]. When faced with cell stresses such as nutrient deprivation or hypoxia, autophagy is important to cellular survival by supplying amino acids recycled from degraded or damaged proteins, as well as by clearing aggregate-prone proteins and damaged organelles, such as mitochondria [2]. In monocytes, autophagy is essential for survival and differentiation and is needed for bacterial and viral agent clearance [3–7].

Macroautophagy, hereafter referred to as autophagy, is a degradative system that clears cytoplasmic organelles and proteins via autophagosomes fused with lysosomes for degradation [8]. The classic model for autophagy is derived from starvation-induced autophagy where the autophagic machinery is thought to be regulated by a class III phosphatidylinositol 3-kinase (PI3K) complex of which beclin1 is a key player [2, 9, 10].

However, several recent studies have shown that autophagy-associated proteins such sequestosome 1 (p62/SQSTM1) also play a role in selective autophagy induced by specific stressors such as misfolded polyubiquitinated proteins, intracellular microbes, and old/damaged mitochondria [11]. In these situations, p62 acts as a mediator in the formation of large aggregates/inclusions known as p62 bodies or sequestosomes [12], which recruit and transport ubiquitinated cargo to the autophagosome for degradation, and this occurs via the association of p62 with the autophagosomal membrane constituent, LC3II (microtubule-associated protein 1 light chain 3) [13–15]. The importance of p62 in liver autophagy was shown by studies in autophagy-deficient mice, which accumulated p62 in hepatocytes with resulting hepatocyte hypertrophy, severe hepatomegaly, and liver injury [16]. Similarly, if p62 is genetically knocked-out, then liver injury caused by impaired autophagy is significantly attenuated [1].

Toll-like receptors (TLRs) are pattern recognition receptors (PRRs) that recognize microbial components and trigger innate immune defenses [5, 6, 12]. TLR4 responds specifically to lipopolysaccharide (LPS), a membrane component of Gram-negative bacteria such as *Escherichia coli* and hepatocytes express and activate TLR4 in response to LPS [17–19]. Past studies have suggested a link between TLR-signaling and autophagy in macrophages and other cell types [6, 7, 10, 20, 21] via activation of downstream signaling pathways [22, 23]. However, little is known of the mechanisms of TLR4-regulation of autophagy in hepatocytes, where autophagy pathways have been shown to be different from macrophages [24] but play an important role in cell health [25].

In this study we investigated the signaling pathways involved in LPS-mediated autophagy in hepatocytes and show that LPS stimulation induced an autophagy-like clearance process in hepatocytes that was dependent on p62 upregulation and not on classical autophagy regulator beclin1.

## 2. Materials and Methods

### 2.1. Cell Culture

Hepatocyte isolation from mice was performed as previously described [18, 26]. Hepatocyte purity was assessed to be >99% by flow cytometry with a typical viability of over 95% using trypan blue staining. Hepatocytes (150,000 cells/mL) were plated gelatin-coated culture plates and cultured in Williams medium E with 10% calf serum, 15 mM HEPES, 10^−6^ M insulin, 2 mM l-glutamine, 100 U/mL penicillin, and 100 U/mL streptomycin. Hepatocytes were allowed to adhere overnight on culture plates. Media was changed to serum-free media before adding treatments and all cells were kept at 37°C in a humidified incubator containing 5% CO_2_.

### 2.2. Reagents and Treatment

Ultrapure LPS (*Escherichia coli* 0111:B4) was purchased from List Biological Laboratories (Vandell Way, CA). siRNA knockdown was accomplished by transfecting plated cells with siRNA for p62, Nrf2, beclin1, MyD88, and TIRAP (Thermo Scientific ON-TARGET plus SMARTpool) with Lipofectamine 2000 (Invitrogen) in OPTI-MEM media (Gibco by Life Technologies) for at least 24 h. Cell media was changed to antibiotic-free media prior to adding siRNA. EBSS media were purchased from Invitrogen.

### 2.3. Animals

Experimental protocols were approved by the Institutional Animal Care and Use Committee of the University of Pittsburgh. All experimental procedures were conducted in accordance with all regulations regarding the care and use of experimental animals as published by the National Institute of Health. Animals were maintained in laminar flow cages in a specific pathogen-free environment at the University of Pittsburgh (Pittsburgh, PA, USA) animal facilities and given standard diet and water. Male wild type C57BL/6 mice were purchased from Jackson Laboratory (Bar Harbor, ME, USA), TLR4KO mice were generated in our laboratory on a C57BL/6 background and backcrossed at least 6 times, and MyD88KO mice on a C57BL/6 background were a generous gift from R. Medzhitov (HHMI, Yale University, New Haven, CT, USA). All animals were allowed access to rodent chow and water *ad libitum*. Mice were between 8 and 10 weeks old.

### 2.4. Cell Lysis and Immunoblot Analysis

Primary mouse hepatocytes were washed with cold PBS, collected in lysis buffer (Cell Signaling Technology, Danvers, MA), sonicated, and centrifuged (13,000 rpm for 15 min), and then the supernatant was collected. Protein concentrations were determined using the bicinchoninic acid (BCA) protein assay kit (Thermo Fisher Scientific, Rockford, IL). Loading buffer was added to the samples and separated by 10% and 15% sodium dodecyl sulfate-polyacrylamide (SDS PAGE) gel. The gel was then transferred to a polyvinylidene fluoride membrane at 250 mA for 2 h. The membrane was blocked in 5% milk for 1 h and then incubated in primary antibody with 1% milk overnight. Membranes were washed in TBS-T (Tween) for 10 min and then placed in horseradish peroxidase secondary antibody for 1 h and washed for 1 h in TBS-T before being developed using a chemiluminescence substance (Thermo Fisher Scientific, Rockford, IL). The primary antibodies used were LC3B, SQSTM1/p62, Beclin-1 (Cell Signaling Technologies, Danvers, MA), LC3B (Novus Biologicals, Littleton, CO), Nrf2 (Santa Cruz Biotechnologies, Dallas, Texas), *β*-actin (Bio-vision, Milpitas, CA), and Myd88 and TIRAP (eBioscience, San Diego, CA). An ubiquitin enrichment kit from Pierce (Thermo Fisher Scientific, Rockford, IL) was used for ubiquitin immunoprecipitation and we followed the manufacturer's protocol.

### 2.5. Gelshift/NF*κ*B EMSA

The nuclear protein fractions were isolated by washing cells with cold PBS twice, collected in Buffer A (10 mM HEPES (pH 7.9), 10 mM KCl, 1.5 mM MgCl_2_, 0.5 mM dithiothreitol (DTT), 0.2 mM PMSF, 0.5% NP-40), incubated on ice for 15 min, centrifuged (5000 rpm for 5 min), discarded supernatant, washed with Buffer A and centrifuged a second time, incubated for an hour on ice with Buffer C + D (20 mM HEPES (pH 7.9), 10 mM KCl, 1.5 mM MgCl_2_, 0.5 mM dithiothreitol (DTT), 0.2 mM PMSF, 10% glycerol, 0.2 mM EDTA), and centrifuged at 13000 rpm for 15 min, and then supernatants were collected. Protein concentration was determined by BCA protein assay according to manufacturer's protocol (Pierce). Double-stranded NF*κ*B specific oligonucleotide was end-labeled by incubation with [^32^P] ATP using T4 polynucleotide kinase (US Biochemicals, Cleveland, Ohio) at 37°C for 30 min and purified through a G-50 Sephadex column at 3000 rpm for 5 min. The nuclear proteins were incubated with 5x binding buffer (Pierce), 1 *μ*g poly (dI-dC), 50,000 cpm of ^32^P-labeled oligonucleotide for 30 min at room temperature. The complexes were run on a 4% nondenatured SDS PAGE gel for 1.5 h in 0.5% Tris-borate-EDTA (TBE) solution, vacuum dried in a gel dryer, and exposed to Kodak X-ray film overnight at 80°C.

### 2.6. Comparative PCR

Total RNA was extracted using RNeasy miniextraction kit from Qiagen (Valencia, CA) according to the manufacturer's instructions. Using 1 *μ*g RNA and oligo dT primers (Qiagen, Valencia, CA) and Omniscript reverse transcriptase (Qiagen, Valencia, CA), cDNA was generated and used for real-time reverse transcription-PCR (RT-PCR). SYBR Green PCR master mix (PE Applied Biosystems, Foster City, CA) was used to prepare the PCR reaction mixes. A two-step real-time RT-PCR was performed using forward and reverse primer pairs prevalidated and specific for p62 (Qiagen, Valencia, CA). All samples were run in triplicates and normalized to *β*-actin mRNA expression.

### 2.7. GFP Transfection and Immunofluorescence

Hepatocytes were plated on coverslips (BD, San Jose, CA) and allowed to adhere on coverslips for 3-4 h before being transfected with adenoviral EGFP-LC3 overnight in Williams medium E (10% calf serum, 15 mM HEPES, 10^−6^ M insulin, 2 mM l-glutamine, 100 U/mL penicillin, and 100 U/mL streptomycin), fixed with 2% paraformaldehyde for 15 min, permeabilized with 0.1% Triton X-100 in PBS for 15 min, blocked with 2% bovine serum albumin (BSA) for 45 min, and washed with cold PBS and 0.5% BSA repeatedly. The cells were incubated with anti-p62 (MBL) or Nrf2 (Novus Biologicals, Littleton, CO) antibodies in 0.5% BSA for 1 h and incubated with goat anti-rabbit IgG conjugated with Cy3 from Invitrogen Life Technologies. DAPI was used to stain the antibodies and then the coverslips were mounted on to glass slides using gelvatol. Autophagic flux was assessed by increase in LC3 puncta in hepatocytes after treatment with bafilomycin (50 nM, Sigma) for 1 h. The cells were imaged using Olympus Fluoview 500 confocal microscope and the fluorescence or LC3II + puncta was quantified using MetaMorph (Molecular Devices, Downingtown, PA) for ≥100 cells.

### 2.8. Statistical Analysis

Data was analyzed for significance by Student's *t*-test using SigmaStat (SPSS, Chicago, IL) and presented as mean ± standard deviation (SD). A *P*-value of < 0.05 was considered significant.

## 3. Results

### 3.1. LPS Stimulates an Autophagy-Like Process in Liver and Hepatocytes

**Figure 1:**
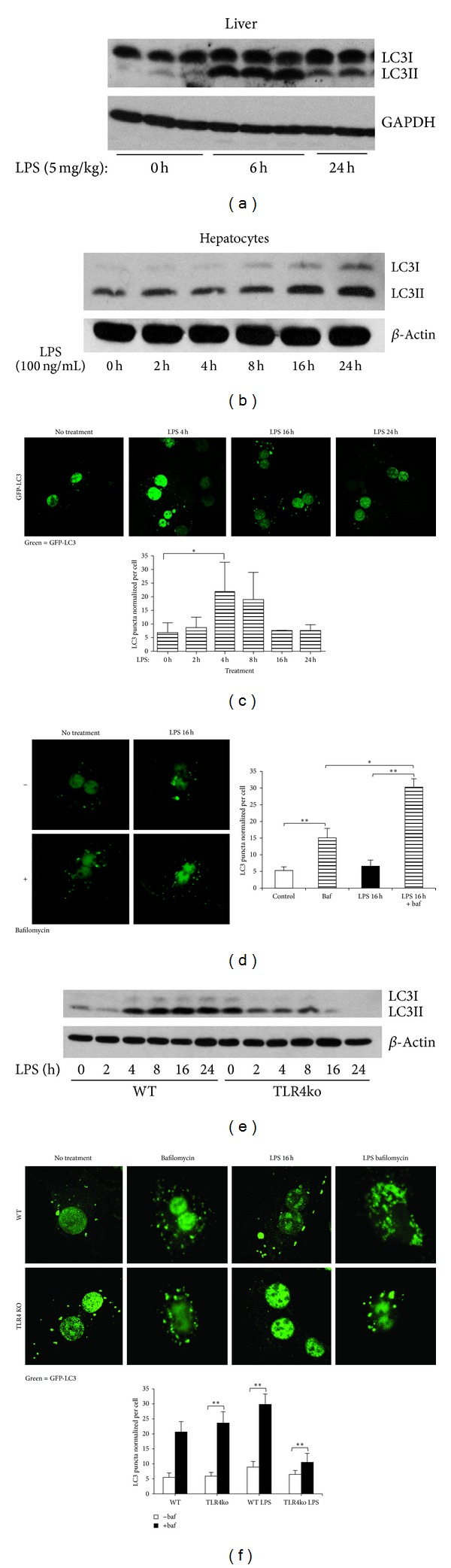
LPS induces TLR4-mediated increases in autophagy in liver and hepatocytes. (a) Immunoblot for LC3II in liver lysates from WT (C57BL/6) mice given no LPS (0 h) or LPS 5 mg/kg ip for 6 or 24 h. Each lane represents liver from one mouse. (b) Immunoblot of LC3II in whole cell lysates from primary mouse hepatocytes treated with LPS (100 ng/mL) for up to 24 h (*n* = 3/gp). (c) LC3-puncta formation with quantification (below) in primary mouse hepatocytes transfected with GFP-LC3 prior to stimulation with LPS (100 ng/mL) for up to 24 h. (d) LC3-puncta formation with quantification (below) in primary mouse hepatocytes pretreated with or without bafilomycin for 1 h plus transfection of GFP-LC3 prior to stimulation with LPS for 16 h. (e) Immunoblot for LC3II in hepatocytes from WT and TLR4ko mice treated for up to 24 h with 100 ng/mL LPS. (f) LC3-puncta formation with quantification (right hand graph) in WT and TLR4ko primary hepatocytes transfected with GFP-LC3 without LPS (0 h or no treatment) and stimulated with LPS (100 ng/mL) for 16 h. **P < 0.01 versus WT LPS 16 h; data shown as mean ± SEM; images representative of at least 3 separate experiments. Confocal immunofluorescence ×60 magnification, quantification using MetaMorph of GFP-positive puncta ≥100 cells normalized to numbers of nuclei.

We first wanted to determine whether LPS induced autophagy in hepatocytes via TLR4 activation. To do this we investigated LC3II expression and puncta formation, a standard method of assessing autophagy [27], in liver and hepatocytes at time points after LPS treatment *in vitro* and *in vivo*. Wild type C57BL/6 (WT) mice were injected intraperitoneally (IP) with LPS (5 mg/kg), and livers were harvested after 6 or 24 h. Whole cell liver lysates then underwent immunoblot analysis. We found that LC3II protein expression in liver increased significantly compared with baseline (0 h; no LPS) and peaked at 6 h (Figure 1(a)) suggesting that autophagy increased in liver after LPS *in vivo*. Similarly, primary hepatocytes isolated from WT mice and treated with LPS (100 ng/mL) in a time-course up to 24 h also showed increased LC3II protein expression over time (Figure 1(b)) suggesting LPS-mediated upregulation of autophagy in hepatocytes *in vitro*.

Autophagosome formation has been shown to correlate with the formation of LC3II-positive puncta viewed fluorescently as aggregate dot-like structures [27]. We transfected WT hepatocytes with GFP-tagged LC3-II (GFP-LC3-II), imaged using confocal microscopy, and measured numbers of LC3II-positive puncta using MetaMorph software. LPS induced statistically significant increases in numbers of LC3II puncta in hepatocytes at 4 h compared with baseline (0 h; no LPS) (Figure 1(c)). In order to verify that autophagic flux was increased we repeated the experiments using bafilomycin to prevent fusion of autophagosomes with lysosomes. Similarly to our results above, Figure 1(d) shows that LPS induces increased autophagic flux as measured by accumulating LC3-positive puncta in hepatocytes after 16 h.

We next wanted to verify that LPS-induced autophagy is TLR4-dependent. Primary hepatocytes were isolated from WT and TLR4ko mice and treated as before with LPS for time points up to 24 h. As expected, LC3-II protein levels did not increase after LPS in TLR4ko cells, compared with WT (Figure 1(e)). Similarly, there were significantly fewer LC3II puncta in TLR4ko cells at 16 h after LPS compared with WT hepatocytes (Figure 1(f)). Taken together, these results indicate that LPS induces an autophagy-like clearance process via TLR4-signaling in primary hepatocytes.

### 3.2. LPS-Induced Autophagy Is Independent of Beclin1

**Figure 2:**
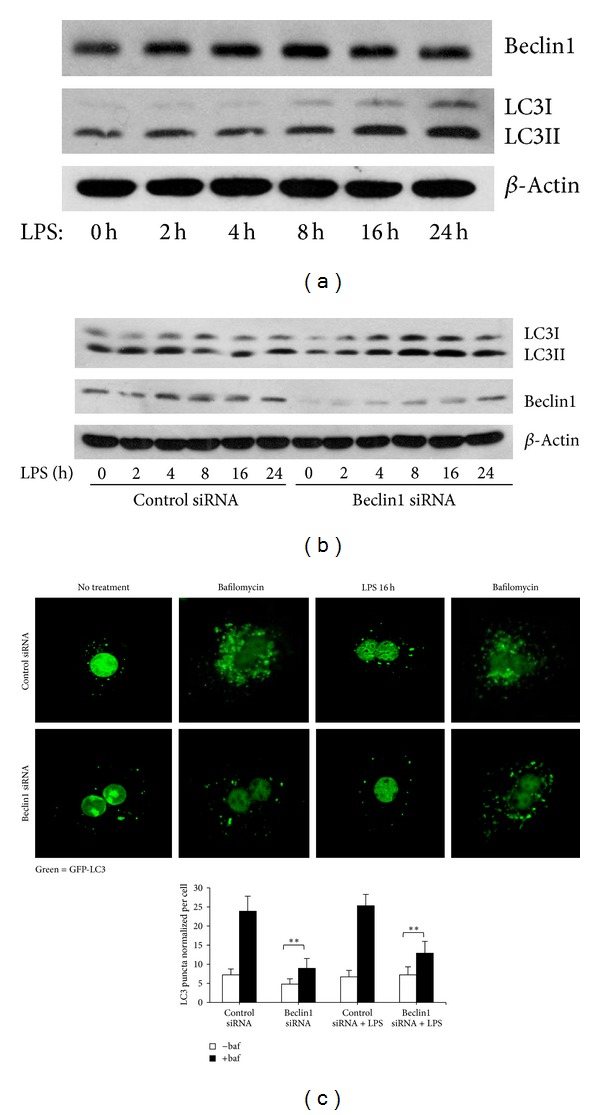
LPS-mediated autophagy is independent of beclin1. (a) Immunoblot for beclin1 and LC3II expression in whole cell lysates from WT hepatocytes without treatment (0 h) or treated with LPS (100 ng/mL) for up to 24 h. (b) Immunoblot for beclin1 and LC3II in whole cell lysates from WT hepatocytes transfected with nontargeting, scrambled (control) siRNA, or siRNA targeting beclin1 and then treated with LPS (100 ng/mL) for up to 24 h. (c) Confocal immunofluorescence of GFP-LC3 in WT mouse hepatocytes transfected with GFP-LC3 and either control or beclin1 siRNA and treated with LPS (100 ng/mL) for 16 h. Data represents mean ± SEM; **P ≤ 0.01 versus siRNA control (time 0) level. Images representative of at least 3 separate experiments. 60x magnification with 1.5x zoom. LC3 puncta was quantified and normalized by number of nuclei for ≥100 cells.

Next, to evaluate whether the observed autophagic-like pathway was initiated through classical autophagy pathways regulated by beclin1, we investigated beclin1 expression in WT hepatocytes after LPS treatment. There was no significant increase in beclin1 protein expression in hepatocytes after LPS treatment (Figure 2(a)). Knockdown of beclin1 also did not prevent the increase in LC3II protein expression after LPS (Figure 2(b)), or the accumulation of LC3-positive puncta within hepatocytes (Figure 2(c)) compared to WT cells treated with control scrambled nontargeting siRNA. These data suggest that LPS-induced autophagy and increased autophagic flux in hepatocytes is independent of beclin1.

### 3.3. LPS Induces Accumulation of p62 Aggregates in Hepatocytes

**Figure 3:**
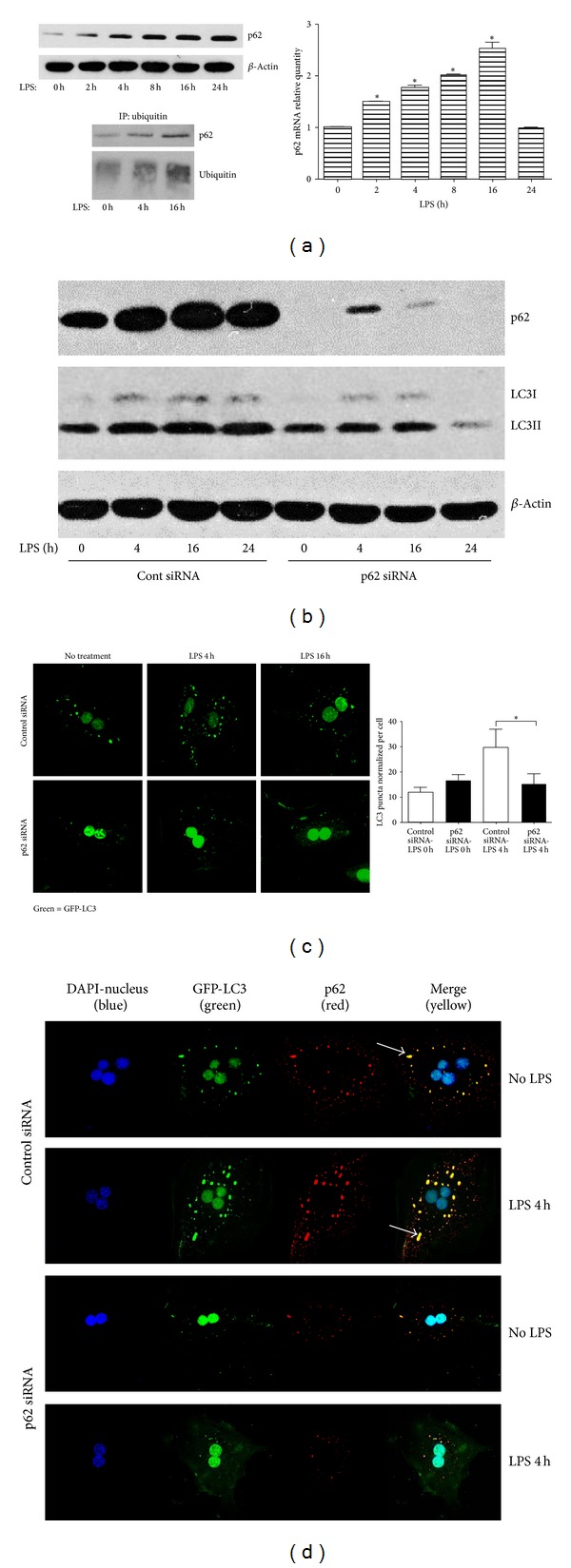
LPS-induced autophagy is dependent on p62. (a) WT hepatocytes were treated with LPS (100 ng/mL) from 2 h to 24 h. p62 protein levels were determined by western blot analysis (left upper image) and mRNA levels were quantified using RT-PCR (right upper image). Data represents mean ± SEM; ∗P ≤ 0.05 versus control (time 0) level. Whole cell lysates from LPS-treated WT hepatocytes were also immunoprecipitated with anti-ubiquitin antibody and then immunoblotted for p62 and polyubiquitin (lower image). (b) Immunoblots for p62 and LC3II in whole cell lysates from WT hepatocytes pretreated with either control siRNA or siRNA targeting p62 followed by LPS stimulation (100 ng/mL) for up to 24 h. (c) Confocal immunofluorescence of GFP-LC3 puncta with quantification (right hand graph) in WT hepatocytes pretreated with control or p62 siRNA followed by LPS stimulation (100 ng/mL) for up to 16 h. (d) Confocal immunofluorescence of GFP-LC3 (green) with p62 (red) showing colocalization (merge = yellow) in WT hepatocytes pretreated with control or p62 siRNA prior to stimulation with LPS (100 ng/mL) for up to 4 h. Arrows indicate p62/LC3 aggregates. Data represents mean ± SEM; *P ≤ 0.05 versus control siRNA LPS 4 h. Images representative of at least 3 separate experiments. Confocal images of 60x magnification with 1.5x zoom. LC3 puncta quantified for ≥100 cells and data normalized by number of nuclei.

SQSTM1/p62 is a multifunctional adaptor protein that recruits ubiquitinated proteins and organelles to LC3II to be taken into the autophagosome and broken down after lysosome fusion [1, 28]. Degradation of p62 and decreased protein levels are thus often used as a marker of autophagy. However, we found that expression of both p62 mRNA and protein increased in hepatocytes in response to LPS (Figure 3(a)). Additionally, immunoprecipitation using an antibody recognizing poly-ubiquitinated proteins confirmed that LPS induces increased p62-ubiquitin association (Figure 3(a), lower).

In order to investigate whether p62 was involved in autophagosome clearance, we knocked-down p62 using siRNA. Knockdown of p62 prevented the LPS-induced increase in LC3II protein expression (Figure 3(b)), as well as significantly preventing the formation of GFP-LC3II puncta after 4 or 16 h of LPS treatment when compared to cells given control nontargeting siRNA (Figure 3(c)). Similarly, knockdown of p62 prevented the colocalization of larger aggregates with LC3-II after LPS (Figure 3(d), arrows) and reduced overall numbers of GFP puncta. Collectively, these results suggest that LPS-induced autophagy is dependent on p62 colocalization with LC3.

### 3.4. LPS-Induced Autophagic-Like Pathway Is Nrf2-Mediated

**Figure 4:**
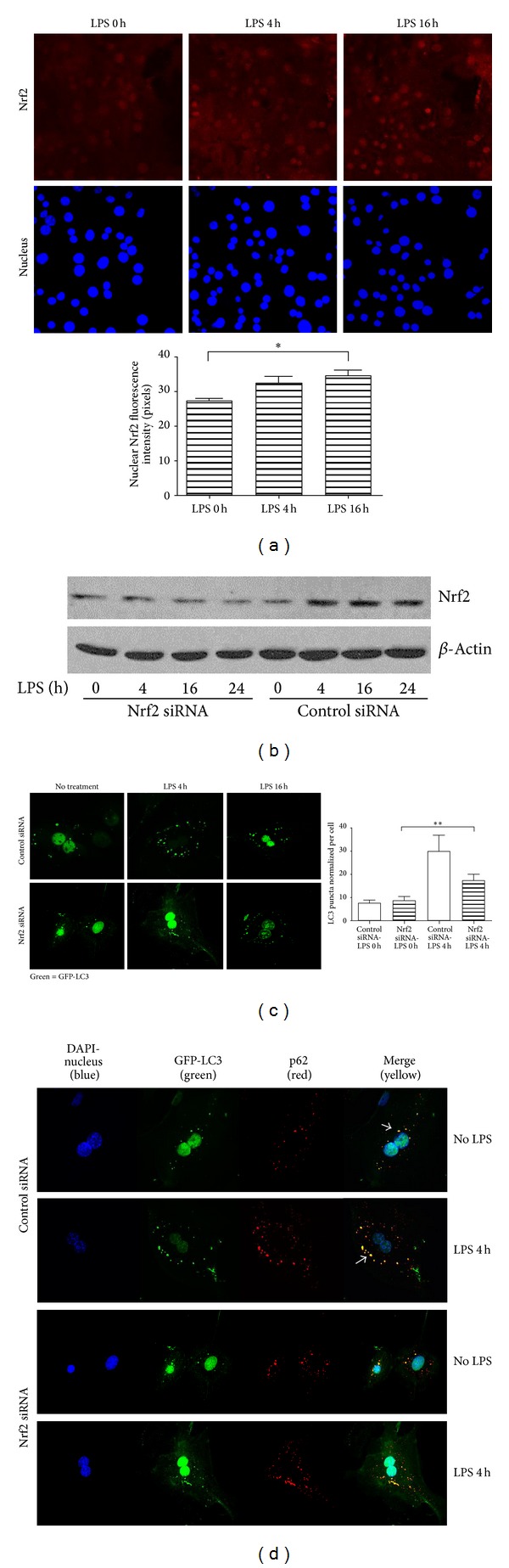
Nrf2-mediates p62-LC3 aggregate formation in hepatocytes. (a) Confocal imaging of Nrf2 (red) and quantification of Nrf2 fluorescence in the nucleus (right hand graph). Data represent mean ± SEM; *P < 0.05 versus control (time 0) level. (b) Immunoblot for Nrf2 in whole cell lysates from WT hepatocytes pretreated with either control siRNA or siRNA targeting Nrf2 followed by LPS stimulation (100 ng/mL) for up to 24 h. (c) Confocal imaging and quantification of GFP-LC3II puncta (right hand graph) in WT hepatocytes pretreated with control or Nrf2-siRNA prior to LPS stimulation for up to 16 h with LPS (100 ng/mL). (d) Confocal immunofluorescence of GFP-LC3 (green) with p62 (red) showing colocalization (merge = yellow) in WT hepatocytes pretreated with either control or Nrf2 siRNA prior to LPS stimulation for 4 h. Data represent mean ± SEM; **P < 0.01 versus Nrf2 siRNA LPS control (time 0); images representative of at least three separate experiments. Confocal microscopy at 60x magnification and 1.5x zoom. Arrows indicate p62/LC3 aggregates. LC3 puncta count was normalized by number of nuclei.

Nrf2 (nuclear factor (erythroid-derived 2)-like 2) is a transcription factor regulated by its own degradation in the cytosol, and activation of which is known to be in part regulated by p62 interactions with an Nrf2-associated protein, Keap1 [4, 29, 30]. Under basal conditions, Nrf2 is degraded, but under conditions of autophagy deficiency, and other stresses, it translocates to the nucleus and upregulates the transcription of multiple proteins including p62 [4, 29]. Therefore, we hypothesized that LPS-induced transcriptional upregulation of p62 in hepatocytes may involve Nrf2. After treating cells with LPS and staining for Nrf2, we saw a modest but statistically significant upregulation of translocation of Nrf2 from the cytoplasm to the nucleus at 16 h (Figure 4(a)). Moreover, knockdown of Nrf2 (Figure 4(b)) using siRNA prevented the increase of LC3-II positive puncta induced by LPS after 4 h (Figure 4(c)). Immunofluorescence also revealed less p62 expression in Nrf2-knockdown cells, decreased p62-LC3 colocalization, and fewer large p62 aggregates compared to the control-treated cells (Figure 4(d), arrows). Similarly, p62 mRNA levels were decreased in Nrf2-knockdown cells (data not shown). These results suggest that Nrf2 partially regulates p62 expression and this, in turn, regulates LPS-induced autophagy in hepatocytes.

### 3.5. MyD88, TIRAP, and NF*κ*B Mediate p62 Upregulation in Hepatocytes

**Figure 5:**
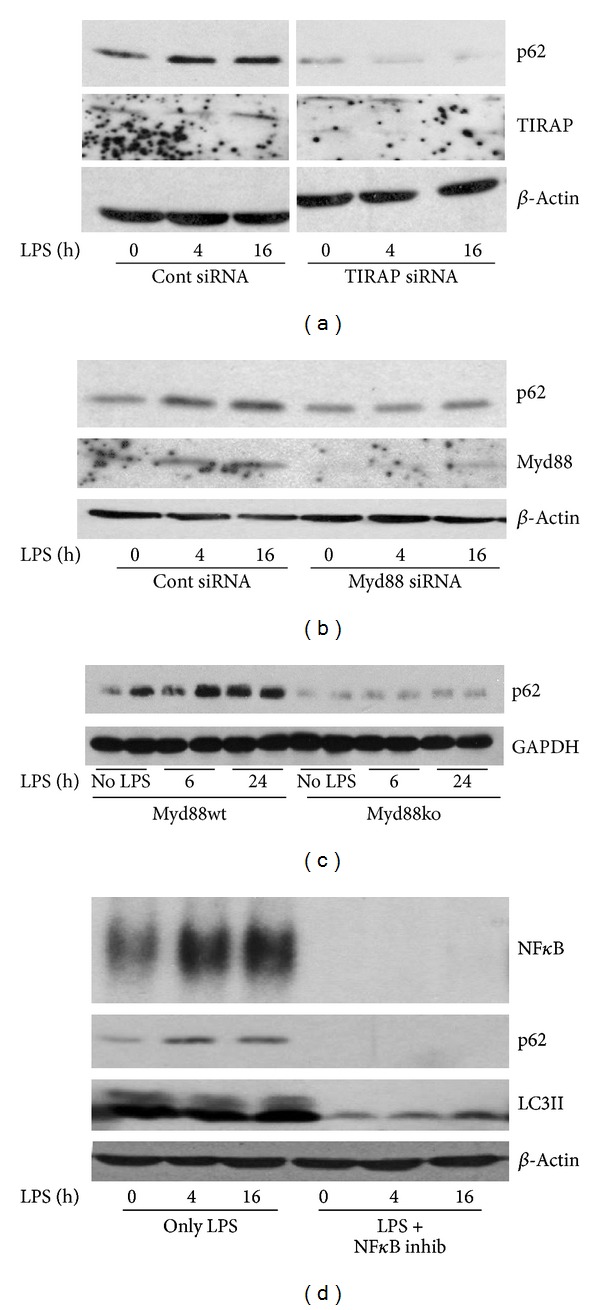
LPS-induced p62-mediated autophagy involves signaling via MyD88/TIRAP and activation of NF*κ*B. (a) Immunoblot of p62 in whole cell lysates from WT hepatocytes pretreated with control or TIRAP-siRNA prior to stimulation with LPS (100 ng/mL) for time points up to 16 h. (b) Immunoblot of p62 in whole cell lysates from WT hepatocytes pretreated with control or MyD88-siRNA prior to stimulation with LPS (100 ng/mL) for up to 16 h. (c) Immunoblot of p62 in whole cell liver lysates from MyD88WT or MyD88ko mice given no LPS, or LPS (5 mg/kg, ip) for 6 or 24 h. Each lane represents liver from one mouse (*n* = 2/gp). (d) WT hepatocytes were given LPS alone (100 ng/mL) or pretreated with NF*κ*B inhibitor followed by LPS stimulation for up to 16 h. Nuclei were extracted from cells for EMSA for NF*κ*B activation and whole cell lysates were collected from similarly treated groups of cells for p62 and LC3II immunoblot. Images representative of at least 3 separate experiments.

We next wanted to find out which TLR4-mediated signaling pathways were important in inducing p62 expression after LPS treatment in hepatocytes. We therefore knocked-down TLR4 adaptor proteins, TIRAP (TIR-containing adaptor protein), and MyD88 in WT hepatocytes using siRNA and measured p62 protein expression by western blot. Knockdown of either TIRAP or MyD88 prevented LPS-mediated increases in p62 protein expression (Figures 5(a) and 5(b)). Similarly, liver expression of p62 in MyD88ko mice treated with LPS (5 mg/kg, IP) did not increase even after 24 h (Figure 5(c)). These data suggest that MyD88 is a dominant TLR4-mediated signaling pathway that induces p62 expression in hepatocytes. NF*κ*B is one of the main inflammatory mediators of MyD88-signaling and is known to also regulate the transcription of p62 [31, 32]. We therefore investigated whether NF*κ*B activation was required for p62 upregulation in our model. Hepatocytes were pretreated with NF*κ*B inhibitor for 24 h and then stimulated with LPS as above. Levels of both p62 and LC3II expression were clearly suppressed after NF*κ*B inhibition (Figure 5(d)).

## 4. Discussion

During sepsis, cells are subjected to increased redox stresses and increased metabolic demands, all of which can lead to cell and mitochondrial damage, accumulation of aggregate-prone or misfolded proteins, depletion of energy resources, and cell death [24, 33, 34]. Ultimately, cellular dysfunction on a large scale in organs and tissues leads to organ failure, which is a major contributing factor for sepsis-induced mortality in patients [35]. One way cells and tissues try to protect themselves from these types of stresses by increasing autophagy and autophagic-like clearance processes [23, 25]. Increased autophagic flux allows cells to clear misfolded proteins and damaged mitochondria in order to try to maintain a stable cytosolic environment and mitigate against increases in reactive oxygen species [25, 36, 37].

Autophagy plays a vital role in the liver and in hepatocytes both under basal conditions and in situations of cell stress [38]. The liver is extremely metabolically active, and autophagy is important in recycling cellular nutrients, maintaining protein folding quality control and also in maintaining mitochondrial health, which is vital for energy homeostasis [25, 39]. Recent work also suggests that autophagy is a key component of lipid homeostasis and metabolic control [40, 41]. Similarly, autophagy has been shown to be hepatoprotective in alcoholic and nonalcoholic fatty liver disease [42], during hepatic ischemia-reperfusion injury [43], in acetaminophen-induced liver injury [44], and also in development of liver tumors [45]. Understanding the cellular pathway involved in the regulation of hepatocyte autophagy is therefore important, and we show in this current study that autophagy can be induced in hepatocytes via TLR4 signaling.

Hepatocytes are continually bathed in small amounts of LPS derived from gut commensal flora and delivered directly to the liver via the portal blood stream [46]. During situations of sepsis, the liver is the main site of clearance of increased levels of LPS from the blood stream, and we have previously shown that hepatocyte TLR4 plays a vital role in this process [18, 19]. In this study we have now determined that TLR4-signaling in hepatocytes is not only important for uptake of LPS into cells but also on increasing cellular processes such as autophagy that are able to degrade bacterial components, and help hepatocytes maintain homeostasis in the face of a hostile cytokine-filled milieu of infection.

The p62 protein is a known key player in the regulation of autophagy and autophagy-like pathways [30] and is vital in selective-autophagy of ubiquitinated proteins [47]. This is known to be important during sepsis, with bacteria that invade the cytosol of cells becoming polyubiquitinated, recognized by p62, and transported to LC3II-positive autophagosome or removal [48]. It therefore makes logical sense that LPS would stimulate similar pathways involving p62 and LC3 to regulate autophagy in hepatocytes, and this is what we showed in our current study. Classically, p62 has also been shown to regulate activation of the transcription factor Nrf2 through association with the Nrf2-regulating protein, Keap1. Accumulating p62 has been shown to bind to Keap1 and allow Nrf2 to dissociate from this complex and translocate to the nucleus, thereby increasing transcription of factors required for redox homeostasis and inflammation [30, 36]. Interestingly, one of the genes activated by Nrf2 is p62 itself, and upregulation of p62 expression may represent a positive feedback loop to maintain protective responses to cell stress. We observed increased Nrf2 activation in hepatocytes after LPS stimulation, and this was associated with upregulation of p62. Indeed, Nrf2 activation was required for LPS-mediated autophagy-like processes to occur in response to LPS in our model. Further study is required to discern the level of equilibrium between p62 expression and Nrf2 activation, and this may be the key regulating step in this form of autophagic clearance mechanism.

## 5. Conclusions

Understanding how TLR4-mediated autophagy-like degradative processes are regulated in the liver and hepatocytes is important if we are to more fully understand how and why organs such as the liver fail during sepsis. In the future this mechanistic understanding may be converted to a clinical benefit for sepsis patients through innovative therapies to prevent sepsis-induced organ failure.
